# Perception of parents/guardians about self-medication for oral problems in their children: a cross-sectional study in Brazilian parents/guardians

**DOI:** 10.1007/s40368-025-01160-0

**Published:** 2026-01-08

**Authors:** M. O. C. Carvalho, M. F. Perazzo, T. S. Pereira, S. M. Paiva, D. C. O. Coutinho, P. A. Martins-Júnior

**Affiliations:** 1 Department of Pediatric Dentistry, Faculty of Dentistry, Federal University of Minas Gerais (UFMG), Av. Antônio Carlos, 6627, Belo Horizonte, MG 31270-901 Brazil; 2 Department of Morphology, Biological Sciences Institute, Federal University of Minas Gerais (UFMG), Belo Horizonte, MG 31270-901 Brazil

**Keywords:** Analgesics, Child, Oral health, Self medication

## Abstract

**Purpose:**

Children and adolescents represent a group strongly prone to the irrational use of medications without medical control. This study aimed to evaluate the practice of self-medication by parents/guardians due to oral health problems in their children.

**Methods:**

A cross-sectional study was carried out with 108 parents/guardians of children. Participants provided socioeconomic data and answered a self-administered questionnaire about the practice of self-medication related to oral problems in their children. Descriptive and bivariate analyzes were performed (*p* < 0.05).

**Results:**

The participants had a mean age of 38.3 ± 9.1 years and were mostly female (*n* = 86; 79.6%). Almost a third (*n* = 35; 32.4%) of the participants have already self-medicated their children due to oral problems. There was a significant association between having self-medicated the children and having already self-medicated himself (*p* < 0.001) and being in favor of self-medication (*p* < 0.001). The most frequently self-medicated oral problems were symptoms of tooth eruption (*n* = 25; 71.4%), toothache (*n* = 21; 60.0%) and oral/traumatic ulcers (*n* = 21; 60.0%). Temporary pain relief was the main motivator for the practice of self-medication (94.3%). Analgesics (82.9%) were the medicines most used. Almost a third (*n* = 10; 28.6%) of the participants who have already self-medicated their children believe that the practice of self-medication does not bring any problems to the children's health.

**Conclusion:**

Parents/guardians self-medicate their children in cases of oral problems and believe that the practice of self-medication does not harm the child's health.

## Introduction

The rational use of medicines occurs when a patient, after proper guidance from a health professional, receives the medicine in doses and periods adequate to his/her clinical needs (WHO 1987). On the other hand, self-medication occurs when a patient or a guardian administers a medicine on their own, without prior guidance from a health professional, to provide relief from symptoms (Arrais et al. 1997).

Self-medication can be influenced by several aspects, such as economic, political and cultural issues. Such factors are associated with the lack of knowledge about the side effects of medicines, wide availability of products, irresponsible advertising, quality of health care, and difficulty in accessing health services, among others (Pereira et al. 2007; Beckhauser et al. 2010). People can use medicines based on past experiences, use old prescriptions or medicine leftovers, share medicines with other family members, or buy them directly without consulting a health professional (Sen Tunc et al. 2021).

In this context, children and adolescents represent a group strongly prone to the irrational use/administration of medicines (Pereira et al. 2007). Side effects, drug interactions, difficulty and delay in correct diagnosis, as well as drug intoxication are some of the several consequences of self-medication in this group (Fundação Oswaldo Cruz 2015). Despite its relevance, studies on self-medication for oral problems in children and adolescents are still scarce (Sen Tunc et al. 2021). According to some authors, relief of pain or fever are one of the main reasons for self-medication for oral conditions, such as toothache and dental eruption (Baig et al. 2012; Paulino et al. 2019). In addition, self-medication for oral problems can lead to significant adverse effects, negatively impacting general health (Paulino et al. 2019). Studies that analyze the occurrence of self-medication involving children and adolescents are important to better understand the reasons that lead parents/guardians to self-medicate their children, providing essential information to support the development of public policies, for the definition of interventions and health promotion strategies, aiming to prevent self-medication to avoid risks to users and community.

The aim of this study was to evaluate the practice of self-medication by parents/guardians due to oral problems in their children. The hypothesis was that self-medication for oral problems is high in children.

## Materials and methods

The present study conforms to the Strengthening the Reporting of Observational Studies in Epidemiology (STROBE) Statement (Malta et al. 2010).

### Study design and eligibility criteria

A cross-sectional study was carried out at the Federal University of Minas Gerais (UFMG) in Belo Horizonte, Brazil. UFMG is considered a reference center for the treatment of oral problems in children and adolescents. Belo Horizonte is located in the southeast region of Brazil. The city has a territorial area of 331,354 km^2^ and an estimated population of 2,530,701 inhabitants (Instituto Brasileiro de Geografia Estatística 2021).

The study was carried out with parents/guardians accompanying children aged 0 to 12 years who attended the Pediatric Dentistru clinic at UFMG. Parents/guardians who were illiterate and those not fluent in Brazilian Portuguese were excluded.

### Sample size calculation

The sample size was calculated with an error of 5.0, a 95% confidence interval, 80% power and a 25.2% expected prevalence of self-medication (Du and Knopf 2009). The minimum estimated sample was 81 participants. A further 30% was added to compensate for possible losses, resulting in an ideal sample of 106 participants.

### Pilot study

Before the main study, a pilot study was carried out with 15 parents/guardians to evaluate the methodology proposed for the study. During this stage, participants evaluated the questions and answers of the questionnaire and they reported whether they understood the questions and answer options. They were also asked to suggest modifications that would enhance understanding and clarity. Some changes were suggested in the terms and expressions used in the questionnaire, such as the terminology of oral problems. All applicable modifications were incorporated in the final version of the questionnaire. Participants in the pilot study were not included in the main study.

### Data collection

Data were collected between November 2018 and February 2021. Sociodemographic status was determined using a self-administered questionnaire addressing the following data from parents/guardians: age, gender, education level and monthly family income (using the Brazilian monthly minimum wage [BMMW] as the reference and categorized as > three times the BMMW or ≤ three times the BMMW).

The practice of self-medication by parents/guardians due to oral problems in their children was collected using a 13-item self-administered questionnaire that addressed: parents' opinion about self-medication, use of old medical prescriptions, respect for package insert instructions, influences in the decision to self-medicate, reasons that led to the practice of self-medication, oral problem, type of medicine used, how the medicine was obtained, perception of the results of self-medication and the risks. All questions were multiple choice.

### Statistical analysis

Statistical analyzes were performed using Jamovi version 2.0.0 (Jamovi Project 2021). Initially, descriptive analyzes and frequency distributions were performed. Chi-square test or Fisher's exact test was performed to test the association between dependent and independent variables, adopting a significance level of 5%.

## Results

**Fig. 1:**
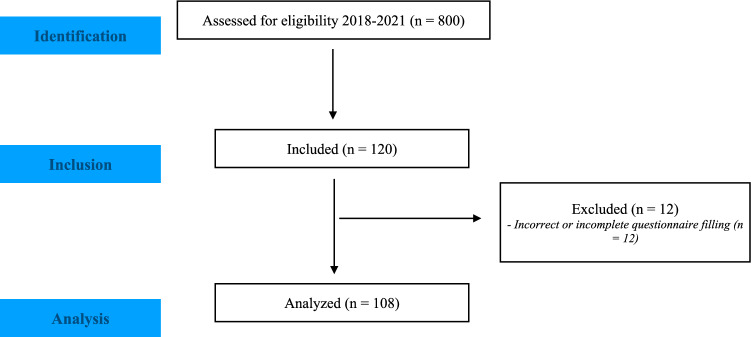
Flowchart

A total of 120 parents/guardians agreed to participate in the study. However, 12 (10.0%) participants were excluded for incorrect or incomplete filling of questionnaires. Then, the final study sample consisted of 108 parents/guardians (Fig. 1). The participants had a mean age of 38.3 ± 9.1 years and were mostly female (*n* = 86; 79.6%), married (*n* = 75; 69.4%) and with more than 8 years of schooling (*n* = 100; 92.6%). Fifty-two (48.6%) participants had a monthly income equal to or lower than three minimum wages (Table 1).

**Table 1:** Sociodemographic characteristics of the participants

Variables	*n* (%)
Gender	
Female	86 (79.6)
Male	22 (20.4)
Marital status	
Married	75 (69.4)
Single	14 (13.0)
Lives with the partner	13 (12.0)
Divorced	6 (5.6)
Occupation	
Employee	47 (43.9)
Autonomous	27 (25.2)
Housewife	20 (18.7)
Unemployed	8 (7.5)
Student	3 (2.8)
Retired	2 (1.9)
Educational level	
≤ 8 years	8 (7.4)
> 8 years	100 (92.6)
Monthly income	
≤ 3 minimum wages	52 (48.6)
> 3 minimum wages	55 (51. 4)
Relationship with the child	
Mother	86 (79.6)
Father	22 (20.4)

More than half of the participants (*n* = 58; 53.7%) declared to have already self-medicated at some point in their lives due to oral problems. Only 17.5% (*n* = 21) of the participants declared themselves in favor of self-medication for oral problems in children. However, almost a third (*n* = 35; 32.4%) have already self-medicated their children due to oral problems at some point in their lives (Table 2). In this sense, there was a significant association between lower monthly income and parental self-medication (*p* = 0.030) and being in favor of self-medication in children (*p* = 0.017).

**Table 2:** Associations between the practice of self-medication due to oral problems in children and independent variables

Independent variables	Have you ever self-medicated your child?		*p*
	No *n* (%)	Yes *n* (%)	
Gender			
Female	17 (77.3)	5 (22.7)	0.277^a^
Male	56 (65.1)	30 (34.9)	
Educational level			
≤ 8 years	5 (62.5)	3 (37.5)	0.712^b^
> 8 years	68 (68.0)	32 (32.0)	
Monthly income			
≤ 3 minimum wages	31 (59.6)	21 (40.4)	0.063^a^
> 3 minimum wages	42 (76.4)	13 (23.6)	
Have already self-medicated?			
Yes	26 (44.8)	32 (55.2)	** < 0.001**^**b**^
No	40 (93.0)	3 (7.0)	
Do not remember/Don't know	7 (100.0)	0 (0.0)	
Is favorable to self-medication?			** < 0.001**^**b**^
Yes	14 (36.8)	24 (63.2)	
No	47 (82.5)	10 (17.5)	
Do not remember/Don't know	12 (92. 3)	1 (7.7)	
Is favorable to self-medication in children?			
Yes	9 (45.0)	11 (55.0)	0.051^b^
No	59 (73.8)	21 (26.3)	
Do not remember/Don't know	5 (62.5)	3 (37.5)	

^a^Chi-square test

^b^Fisher's exact test

Bold values indicate statistical significance (*p* < 0.05)

The child's self-medication was generally based on old medical/dental prescriptions (*n* = 13; 37.1%), although some participants claimed to have been based on old prescriptions from other people (*n* = 7; 53.9%). There was a significant association between child's self-medication due to oral problems and parental self-medication (*p* < 0.001) and being in favor of self-medication (*p* < 0.001) (Table 2).

The most frequently self-medicated oral problems were symptoms of tooth eruption (*n* = 25; 71.4%), toothache (*n* = 21; 60.0%), "traumatic sore/ulcer" (*n* = 21; 60.0%), gum swelling (*n* = 12; 34.3%), "candidiasis" (*n* = 12; 34.3%), tooth mobility (*n* = 8; 22.9%), dental trauma (*n* = 6; 17.1%), bad breath (*n* = 5; 14.3%), "herpes" (*n* = 4; 11.4%) and bleeding gums (*n* = 3; 8.6%). Several medicines were used for this purpose. The most commonly used medicines were analgesics (*n* = 29; 82.9%), anesthetics (*n* = 19; 54.3%) and anti-inflammatory drugs (*n* = 13; 37.1%) (Table 3). The main sources for seeking information about medication were family (*n* = 14; 40.0%), friends (*n* = 13; 37.1%). Health professional (*n* = 13; 37.1%) and pharmacist (*n* = 12; 34.3%) were informed by some parents/guardians, but related to old prescriptions. Medicines were purchased more frequently at the pharmacy (*n* = 32; 91.4%).

**Table 3:** Types of medicines used

Types of medicines	*n* (%)
Analgesics	29 (82.9)
Anesthesics	19 (54.3)
Anti-inflammatory	13 (37.1)
Antipyretics	7 (20.0)
Natural products (Propolis; salt and water)	6 (17.1)
Antifungals	5 (14.3)
Antibiotics	3 (8.6)
Antivirals	3 (8.6)

Participants stated that the main motivator for child's self-medication was temporary pain relief (*n* = 33; 94.3%), with the majority reporting that their children felt better after self-medication (*n* = 33; 94.3%). Almost a third (*n* = 10; 28.6%) of the participants who have already self-medicated their children for oral problems believe that the practice of self-medication does not bring any problems to the children's health.

## Discussion

This study was carried out to assess the practice of self-medication by parents/guardians due to oral problems in their children. The main results of this study suggest that the practice of self-medication is expected and that some participants believe that self-medication does not bring any health problems for children. Thus, it is necessary to develop health promotion strategies aimed at clarifying and raising awareness of the population about the risks related to the practice of self-medication.

### Relevance of the study

Although there are several studies on the practice of self-medication, most of them have been carried out in adults (Arrais et al. 2016; Loyola Filho et al. 2002). In addition, studies carried out in children and adolescents are usually not specific about self-medication for oral problems (Tarciuc et al. 2020; Telles Filho and Pereira Júnior 2013). Addressing the practice of self-medication in children is relevant because pediatric patients are recognized as a special population for drug therapy. This is due to the constant physiological and anatomical changes that occur during childhood, impacting the pharmacokinetics and dynamics of compounds (Batchelor and Marriott 2015). In this sense, the drug dose to be administered to pediatric patients should be adjusted to provide similar internal exposure and pharmacodynamic effects (Batchelor and Marriott 2015). Thus, performing self-medication in children is extremely dangerous, regardless of the purpose, since child intoxication due to medicines can compromise the functioning of organs and generate sequelae (Moraes et al. 2013).

### Perceptions about self-medication

In this study, a small portion of participants reported being in favor of the self-medication of children for oral problems. In a study carried out in a pediatric emergency care center, there was a frequency of 67.2% of parents/guardians in favor of self-medication of children (Nogueira et al. 2015), which is higher than that observed in the present study. The specific circumstances of each study can explain these discrepancies. Research participants by Nogueira et al. (2015) were accompanying their children in an emergency room, in which patients usually have painful symptoms. Therefore, there is a greater probability that parents/guardians have already self-medicated their children prior to the dental appointment and observed symptoms relief.

In the present study, a frequency of 32.4% of self-medication was observed in children with oral problems, with a significant association between parental self-medication and self-medication in children. Other studies on the subject found higher frequencies of self-medication (Paulino et al. 2019; Thikkurissy et al. 2012). In addition, a study carried out during the COVID-19 pandemic found a frequency of self-medication higher than 70% (Sen Tunc et al. 2021). A recent study using Google Trends showed an increase in the number of global self-medication searches since the pandemic's beginning (Onchonga 2020). Since the present study was partially conducted during the COVID-19 pandemic, it is possible that the use of self-medication observed could also be influenced by the pandemic.

On the other hand, a Brazilian study evaluating self-medication in children and pre-adolescents found a frequency of self-medication of 20.9% (De Lima et al. 2016). However, it should be noted that this study assessed patterns of self-medication for dental infections. Furthermore, studies on self-medication for general health problems in children found frequencies ranging between 6.6% (Arrais et al. 2016) and 70% (Tarciuc et al. 2020). Comparisons should be made with caution, as these are studies evaluating the practice of self-medication in other contexts.

Some studies have argued that the practice of self-medication can be influenced by different factors, such as age, gender, education, monthly income and accessibility to the health system (Loyola Filho et al. 2002; Brlić et al. 2014). In the present study, there was a significant association between lower monthly income and parental self-medication and being in favor of self-medication in children. Still, there was no association between the practice of self-medication in children and the gender, educational level and monthly income of the participants, corroborating data from other studies (Pereira et al. 2007; Nogueira et al. 2015; Paulino et al. 2019).

### Reasons for self-medication

Symptoms of tooth eruption, toothache and traumatic cold sore/ulcer were the leading causes for the practice of self-medication in children. In this sense, the temporary relief of pain was the primary motivator for the practice of self-medication. These findings are similar to other studies that showed that toothache is the main trigger for self-medication in children (Paulino et al. 2019; Sen Tunc et al. 2021). Traumatic cold sore/ulcer also causes painful sensation (Johnston et al. 2022), leading to negative impacts on children and adolescents' oral health-related quality of life (Gherunpong et al. 2004). Tooth eruption symptoms can also cause reactions in children; therefore, parents concerned about the symptoms end up self-medicating their children. On the other hand, a study found that non-pharmacological methods such as teething rings, cuddle therapy and rubbing the gums effectively improved tooth eruption symptoms (Memarpour et al. 2015).

### Main medicines used for self-medication

Analgesics were the drugs most frequently used in the practice of self-medication in children, as found in other studies (Stolbizer et al. 2018; Paulino et al. 2019; Bhattarai et al. 2020; Sen Tunc et al. 2021). Accessibility, exemption from using a prescription and low cost can facilitate the use of analgesics. Many parents/guardians believe that these drugs are not toxic or bring little harm to health (Sen Tunc et al. 2021). However, studies point to analgesics as one of the main causes of intoxication in children aged zero to five years (Maior et al. 2017; Matos et al. 2002; Pereira et al. 2013). Hommez et al. (2018) showed that patients treated at the emergency dental service had a high risk of analgesic overdose and patients' lack of knowledge of the maximum daily dose was identified as one of the reasons for overdose.

Anesthetics and anti-inflammatory drugs have also been indicated as the drugs of choice for self-medication in children. A study showed that Ibuprofen or Paracetamol were the most recommended medicines to relieve tooth eruption symptoms (Thompson and Huntington 2019). Paulino et al. (2019) observed that the use of topical anesthetics and anti-inflammatory drugs were chosen for pain relief, but less frequently than analgesics. According to the study by Plutzer et al. (2012), mothers who received information about symptoms commonly associated with tooth eruption and ways to manage them were less likely to use topical and oral medicines and relied more on non-pharmacological techniques, such as rubbing the gums to relieve discomfort. In xxxx, since 2010, antibiotics are only available with a medical prescription, which makes it more difficult to obtain this medicine. This is reflected in the study since only three participants reported the use of antibiotics in the children, being all of them reused leftover antibiotics.

Self-medication can be carried out through the acquisition of medicines without a prescription, the sharing of medicines, the reuse of leftover medicines and the use of old prescriptions (Pereira et al. 2007). In this study, the choice of medicines was mainly based on the recommendation of family members, and many parents/guardians stated that they had already used previous prescriptions. Sen Tunc et al. (2021) found a frequency of 67.7% of parents/guardians who reported the use of medicines previously prescribed for their children and also evidenced the practice of self-medication based on advice from close relatives and pharmacists. This finding was attributed to the difficulty in accessing health services during the COVID-19 pandemic (Sen Tunc et al. 2021), which may also have occurred in the present study. Other studies confirm the reuse of old prescriptions for self-medication and highlight that this occurs more frequently in children under seven years of age (Beckhauser et al. 2010; Telles Filho and Pereira Júnior 2013).

### Limitations

This study has some limitations that should be recognized. The cross-sectional nature of the study made it impossible to assess causality. Also, the convenience sample limits the extrapolation of results to the general population, impacting the generalisability of the study. Therefore, it is important to carry out further studies with larger/representative samples to assess self-medication for oral problems in children.

Despite these limitations, the results of this study are original and relevant, indicating that the practice of self-medication in children is common. Therefore, dentists and health professionals must instruct parents/guardians about the risks of self-medication during appointments. Informational brochures with accessible language can be made available at basic health units, offices, pharmacies, and on the internet. In addition, these results can serve as data to aid in the implementation of public policies to prevent self-medication and to avoid harmful consequences to children.

## Conclusion

Parents/guardians frequently self-medicate their children due to oral problems, especially in situations of toothache and tooth eruption symptoms. Analgesics were the most frequently used medicines. Parents/guardians who self-medicate their children believe that this practice does not harm their health.

## Data Availability

Data will be available upon a reasonable request.
